# Supplementary figures and images for: ProxECAT: Proxy External Controls Association Test. A new case-control gene region association test using allele frequencies from public controls
Source: PLoS Genet. 2018 Oct 16;14(10):e1007591. doi: 10.1371/journal.pgen.1007591 (PMC6191077; doi:10.1371/journal.pgen.1007591)

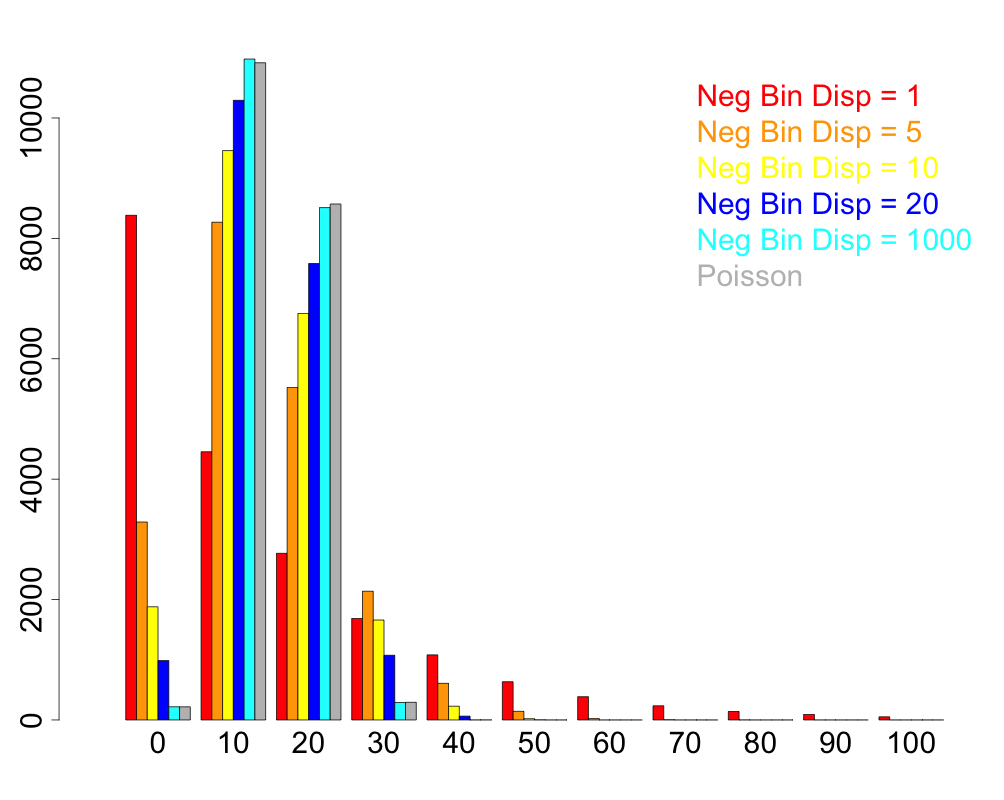

Supplement: S1 Fig — (PNG) [file pgen.1007591.s001.png]

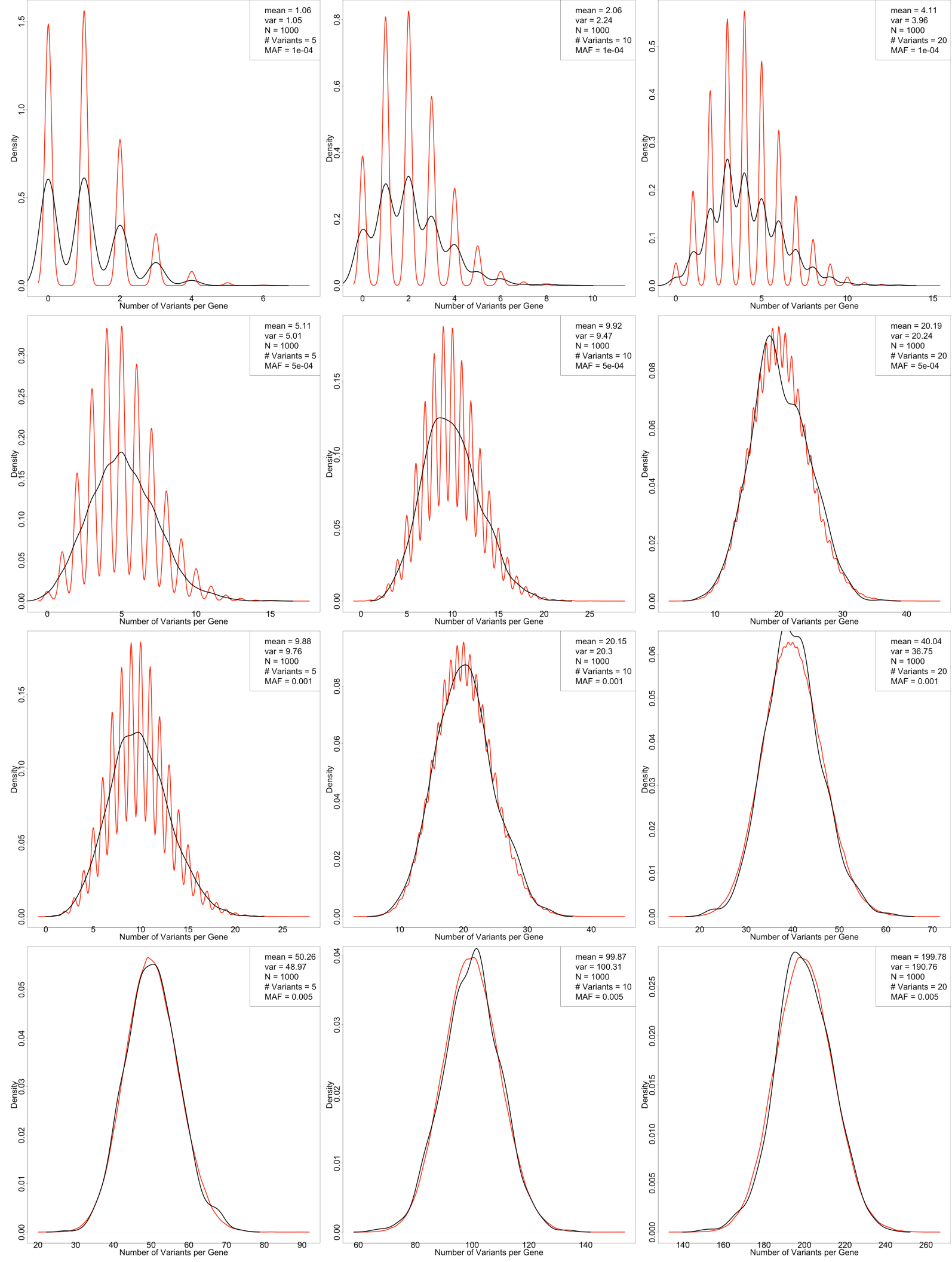

Supplement: S2 Fig — MAF = 0.0001, 0.0005, 0.001, 0.005; number of minor variant alleles within the gene region = 5, 10, 20. (PDF) [file pgen.1007591.s002.pdf]

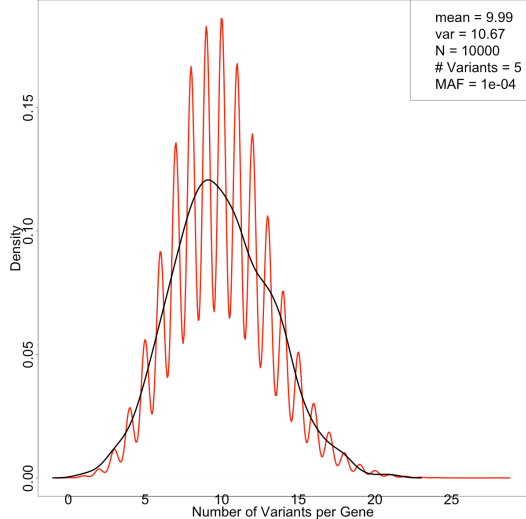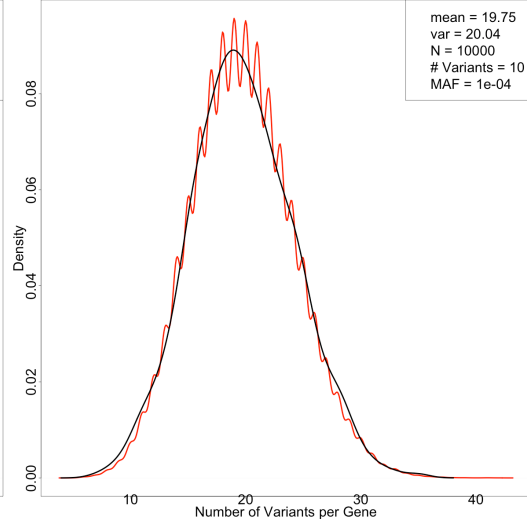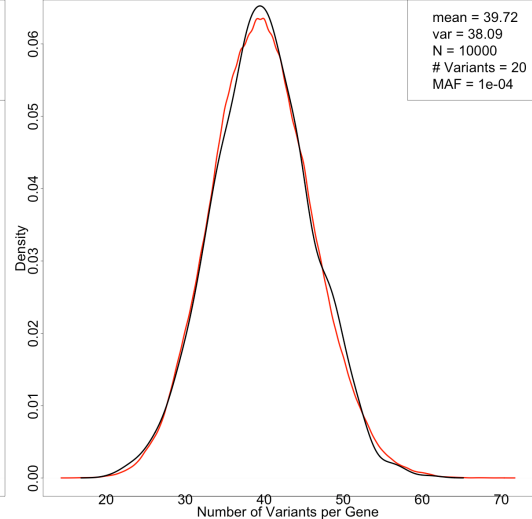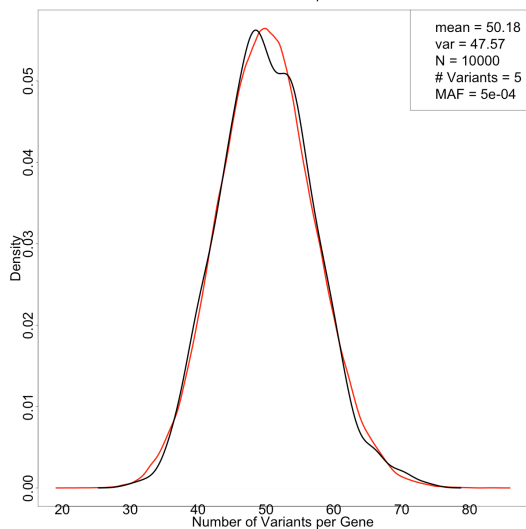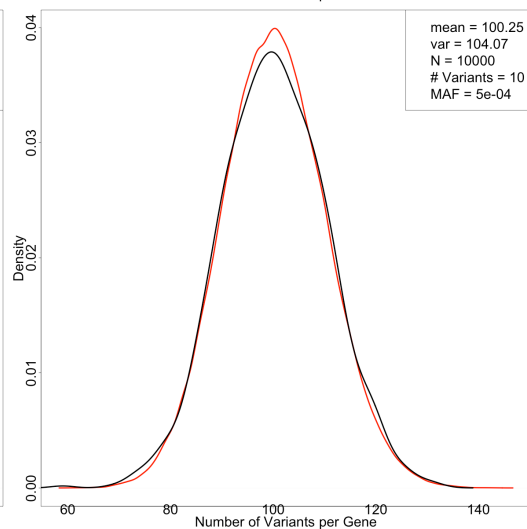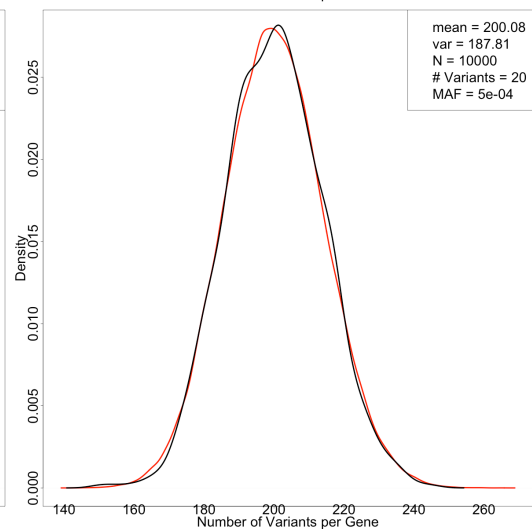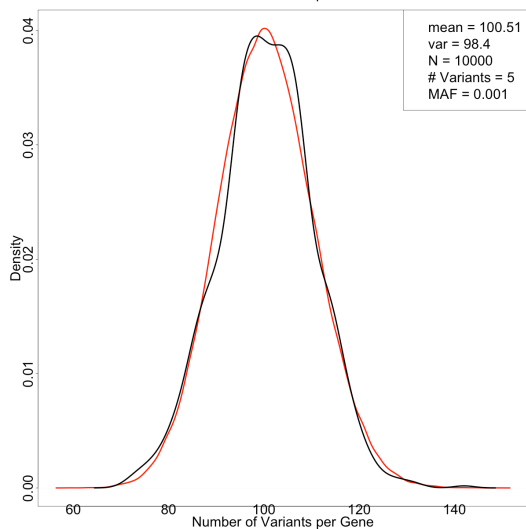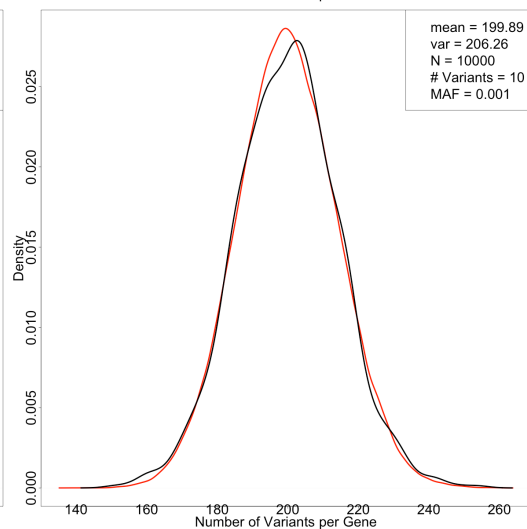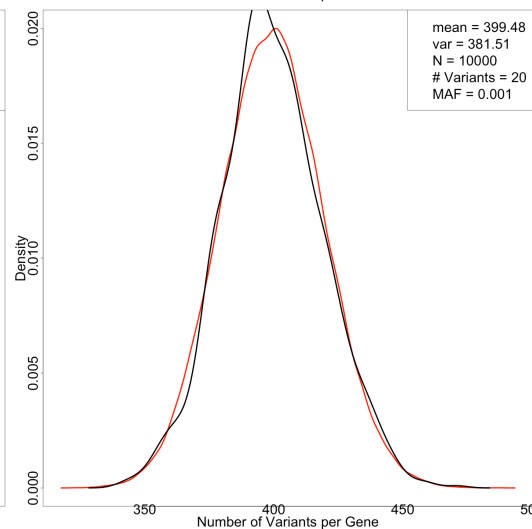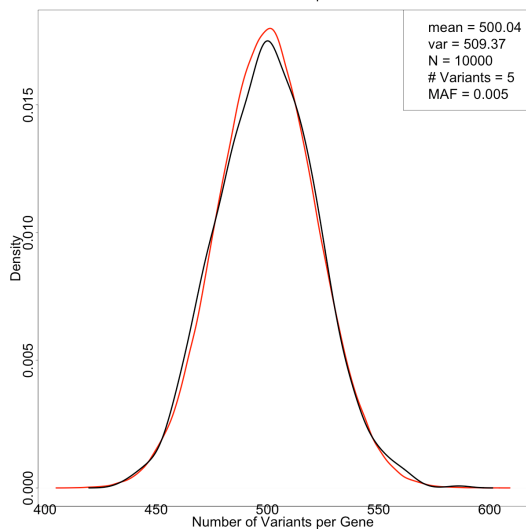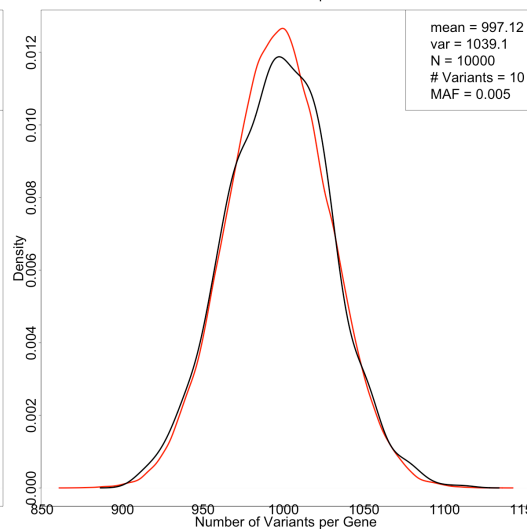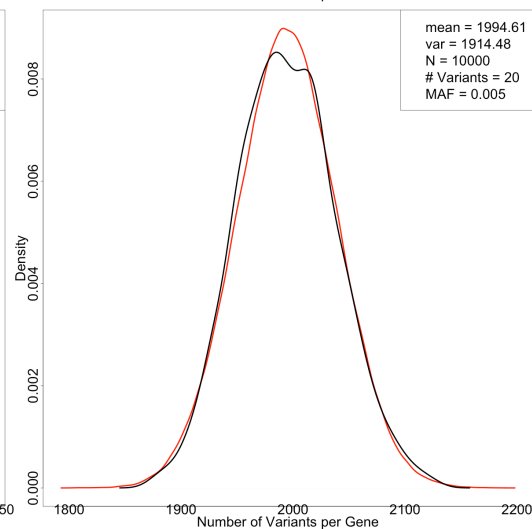

Supplement: S3 Fig — MAF = 0.0001, 0.0005, 0.001, 0.005; number of variants within the gene region = 5, 10, 20. (PDF) [file pgen.1007591.s003.pdf]

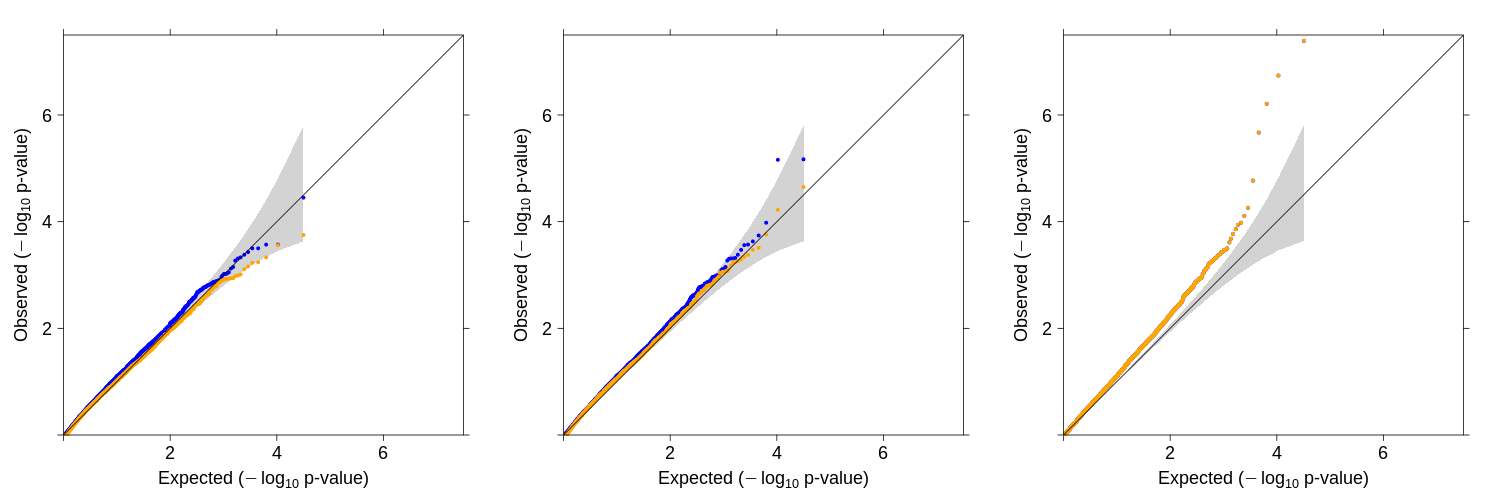

Supplement: S5 Fig — Internal MAF < 0.001 and number of alleles per gene ≥ 5 for functional and proxy. ProxECAT (blue), ProxECAT-weighted (orange), 95% confidence interval of expected results in gray. Left: SYN, Ngenes = 15,779 (ProxECAT lambda = 1.233, ProxECAT-weighted = 1.081). Middle: LOW, Ngenes = 15,874, (ProxECAT lambda = 1.215, ProxECAT-weighted lambda = 1.119). Right: NOT FUNC, Ngenes = 16,011 (ProxECAT lambda = 1.18, ProxECAT-weighted = 1.18). For the NOT FUNC proxy group, the weights for ProxECAT-weighted are one for both cases and controls resulting in identical distributions of test statistics for ProxECAT and ProxECAT-weighted. (PNG) [file pgen.1007591.s005.png]
