# Supplementary material for: ProxECAT: Proxy External Controls Association Test. A new case-control gene region association test using allele frequencies from public controls
Source: PLoS Genet. 2018 Oct 16;14(10):e1007591. doi: 10.1371/journal.pgen.1007591 (PMC6191077; doi:10.1371/journal.pgen.1007591)
Supplement: S4 Fig — Comparison of the natural log of the number of alternate alleles observed in each gene region for functional variants (FUNC) and three proxy selection strategies: synonymous (SYN), low impact (LOW), not functional (NOT FUNC). Top right panels: scatter plots with y = x line. Bottom left panels: correlation coefficient. (PDF) [file pgen.1007591.s004.pdf]

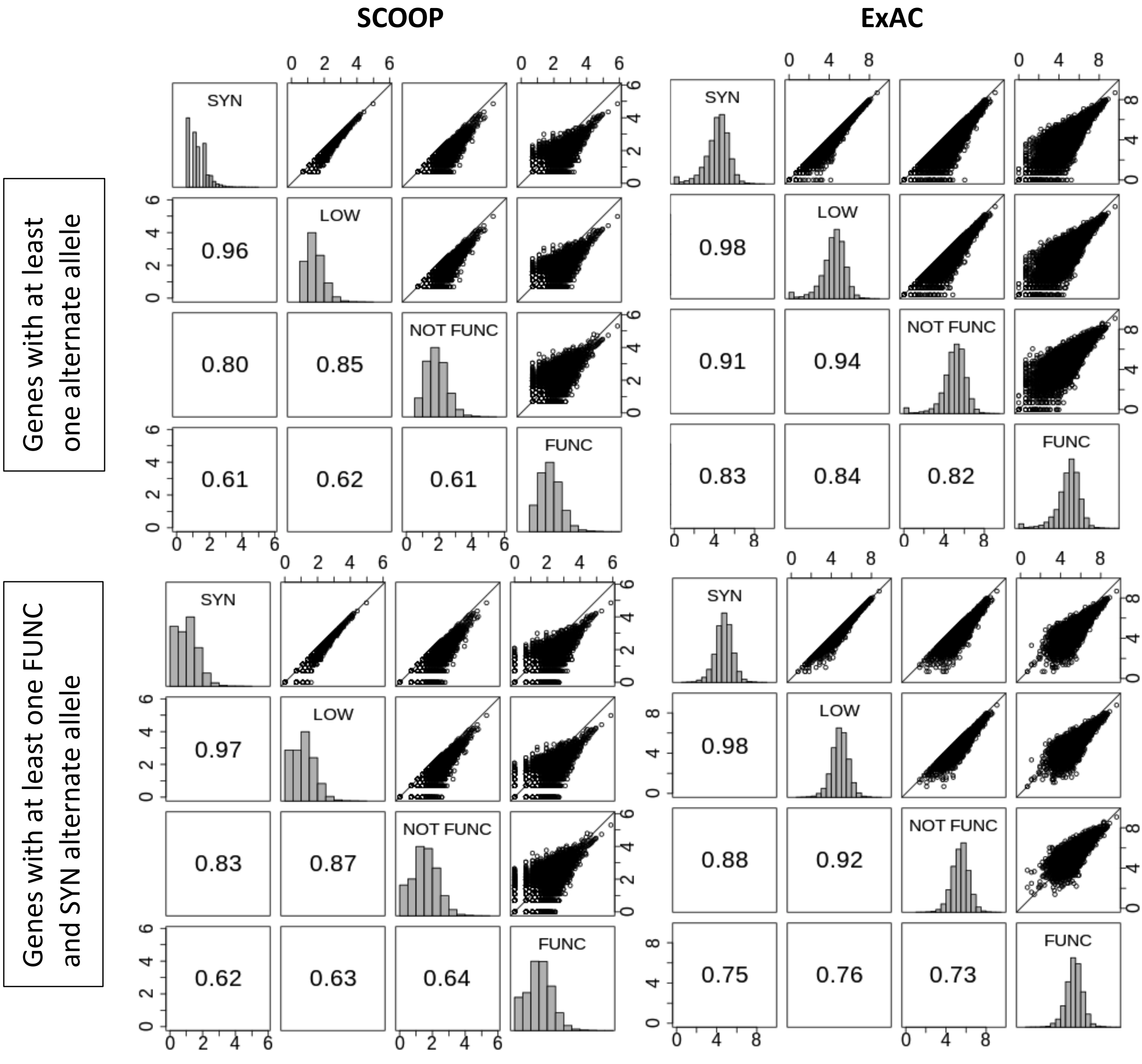

SCOOP
ExAC
Genes with at least one alternate allele
Genes with at least one FUNC and SYN alternate allele
SYN
LOW
NOT FUNC
FUNC
0.96
0.80
0.85
0.61
0.62
0.61
0.98
0.91
0.94
0.83
0.84
0.82
0.97
0.83
0.87
0.62
0.63
0.64
0.98
0.88
0.92
0.75
0.76
0.73
