# Supplementary material for: ProxECAT: Proxy External Controls Association Test. A new case-control gene region association test using allele frequencies from public controls
Source: PLoS Genet. 2018 Oct 16;14(10):e1007591. doi: 10.1371/journal.pgen.1007591 (PMC6191077; doi:10.1371/journal.pgen.1007591)
Supplement: S1 Appendix — (PDF) [file pgen.1007591.s013.pdf]

# Appendix 1

## Derivation of Maximum Likelihood Estimates for ProxECAT

For simplicity in our derivation, we use the following notation for our four groups

**Supplemental Table 1.** Data notation for internal case an external control samples for ProxECAT

| | | Predicted Functional Impact | |
|---|---|---|---|
| | | **Functional** | **Not Functional (Proxy)** |
| **Cases (Internal)** | Y = 1 | $x_1 = x_1^f$ | $x_2 = x_1^p$ |
| **Controls (External)** | Y = 0 | $x_3 = x_0^f$ | $x_4 = x_0^p$ |

Thus, we can write the likelihood as

$$L(\lambda_1, \lambda_2, \lambda_3, \lambda_4) = \prod_{k=1}^{K} \frac{e^{-\lambda_k} {\lambda_k}^{x_k}}{x_k!}$$

We will use the constraint as defined by the null hypothesis and the method of Lagrange Multipliers to find the maximum likelihood estimates of our parameters: $\lambda_1, \lambda_2, \lambda_3, \lambda_4$

$$H_0: \frac{\lambda_1}{\lambda_2} = \frac{\lambda_3}{\lambda_4}$$

We first take the log of both the likelihood and the constraint.

$$\ell(\lambda_1, \lambda_2, \lambda_3, \lambda_4) = -log\left(\prod_{k=1}^{K} x_k!\right) - \sum_{k=1}^{K} \lambda_k + \sum_{k=1}^{K} x_k log(\lambda_k)$$

$$g(\lambda) = log(\lambda_1) - log(\lambda_2) - log(\lambda_3) + log(\lambda_4) = 0$$

After the change of variables $\lambda_k = e^{\omega_k}$, we arrive at the problem

$$max\ f(\underline{\omega}) = c - \sum_{k=1}^{K} e^{\omega_k} + \sum_{k=1}^{K} x_k \omega_k$$

$$\text{Subject to } g(\underline{\omega}) = \omega_1 - \omega_2 - \omega_3 + \omega_4 = 0$$

Noting that the objective function is concave and the constraint is linear, we can obtain the maximum using the Method of Lagrange Multipliers.

Define the Lagrangian function as

$$\mathcal{L}(\underline{\omega}, \mu) = f(\underline{\omega}) + \mu\, g(\underline{\omega})$$

where $\mu$ is the Lagrange multiplier

The optimality conditions are

$$\frac{\partial\mathcal{L}(\underline{\omega},\mu)}{\partial\omega_1} = -e^{\omega_1} + x_1 + \mu = 0$$

$$\frac{\partial\mathcal{L}(\underline{\omega},\mu)}{\partial\omega_2} = -e^{\omega_2} + x_2 - \mu = 0$$

$$\frac{\partial\mathcal{L}(\underline{\omega},\mu)}{\partial\omega_3} = -e^{\omega_3} + x_3 - \mu = 0$$

$$\frac{\partial\mathcal{L}(\underline{\omega},\mu)}{\partial\omega_4} = -e^{\omega_4} + x_4 + \mu = 0$$

Solving for each $\omega_k$, the constraint, $g(\underline{\omega}) = 0$ becomes

$$log(x_1 + \mu) - log(x_2 - \mu) - log(x_3 - \mu) + log(x_4 + \mu) = 0$$

from which we obtain

$$\mu = \frac{x_2x_3 - x_1x_4}{x_1 + x_2 + x_3 + x_4}$$

Thus, for our initial parameters we have the following estimates:

$$\hat{\lambda}_1 = e^{\omega_1} = x_1 + \mu = \frac{x_1^2 + x_1x_2 + x_1x_3 + x_2x_3}{x_1 + x_2 + x_3 + x_4}$$

$$\hat{\lambda}_2 = e^{\omega_2} = x_2 - \mu = \frac{x_2^2 + x_1x_2 + x_2x_4 + x_1x_4}{x_1 + x_2 + x_3 + x_4}$$

$$\hat{\lambda}_3 = e^{\omega_3} = x_3 - \mu = \frac{x_3^2 + x_1x_3 + x_3x_4 + x_1x_4}{x_1 + x_2 + x_3 + x_4}$$

$$\hat{\lambda}_4 = e^{\omega_4} = x_4 + \mu = \frac{x_4^2 + x_2x_4 + x_3x_4 + x_2x_3}{x_1 + x_2 + x_3 + x_4}$$

We can then use the parameter estimates in the likelihood for the constrained null hypothesis. The maximum likelihood estimates for the unconstrained alternative hypothesis are

$$\tilde{\lambda}_1 = x_1$$

$$\tilde{\lambda}_2 = x_2$$

$$\tilde{\lambda}_3 = x_3$$

$$\tilde{\lambda}_4 = x_4$$

Using these estimates in the constrained (i.e. under the null hypothesis) likelihood and unconstrained (i.e. under the alternative hypothesis) likelihood, we can complete a likelihood ratio test as follows

$$\Lambda = LRT(x) = \frac{L(\lambda_{H0}|x)}{L(\lambda_{H1}|x)}$$

which, by Wilk's theorem can be transformed to have a chi-squared distribution as follows:

$$-2log(\Lambda) \sim \chi^2(df = 1)$$
